# Effectiveness of Nephron Sparing Surgery and Radical Nephrectomy in the Management of Unilateral Wilms Tumor: A Meta-Analysis

**DOI:** 10.3389/fonc.2020.01248

**Published:** 2020-09-04

**Authors:** Hongkun Chen, Shuqing Yang, Cheng Qian

**Affiliations:** ^1^Department of Pediatric Surgery, Zaozhuang Municipal Hospital, Zaozhuang, China; ^2^Zaozhuang Hospital of Traditional Chinese Medicine, Zaozhuang, China

**Keywords:** nephroblastoma, nephrectomy, renal tumor, pediatric cancer, renal function

## Abstract

**Background:** Unilateral Wilms tumor is the most common renal malignancy in the pediatric population. Although the onset of surgical intervention like radical nephrectomy has substantially reduced the mortality rate, recent evidence has raised concerns regarding several postoperative complications associated with this procedure. Nephron sparing surgery has been reported to avoid such postoperative complications and have high technical success rate. However, no attempt to date has been made to synthesize the evidence comparing the efficacy of radical nephrectomy and nephron sparing surgery for managing unilateral Wilms tumor.

**Methods and Results:** To metastatistically compare the efficiency of radical nephrectomy with nephron sparing surgery for managing unilateral Wilms tumor, a systematic identification of the literature was performed according to the Preferred Reporting Items for Systematic Reviews and Meta-analyses guidelines on four academic databases: MEDLINE, Scopus, EMBASE, and CENTRAL. A meta-analysis comparing renal function (estimated glomerular filtration rate), survival rate, and rate of relapse was performed to compare the efficacy of radical nephrectomy and nephron sparing surgery. Out of 1,283 records, 20 articles including 5,246 children (mean age, 4.3 ± 3.0 years) were included in this review. Radical nephrectomy was performed on 11 of the included studies, whereas nephron sparing surgery was performed on five studies. Two studies compared the efficacy of both interventions. The meta-analysis reveals the beneficial effects of nephron sparing surgery (Hedge's *g*, 0.76) as compared to radical nephrectomy (−0.16) for the estimated glomerular filtration rate for children with unilateral Wilms tumor. Moreover, higher survivability (0.59) and lesser occurrence of relapse were (−1.0) also reported for cases operated with nephron sparing surgery.

**Conclusion:** The current meta-analysis recommends the use of nephron sparing surgery for unilateral Wilms tumor. The procedure accounts for higher survivability and postoperative renal function and lesser incidence of relapse as compared to radical nephrectomy.

## Introduction

Wilms tumor is the largest cause of renal malignancy in the pediatric population (1–3). The onset of this malignancy begins in the renal parenchymal area possibly by the facilitated proliferation of the residue in mesonephroma (4, 5), after which the tumor grows into a large spherical intrarenal mass in the presence of perirenal fat (circumference, ≥5 cm) (6, 7). Epidemiological studies suggest that the highest occurrence of Wilms tumor happens unilaterally (8), thereby accounting for almost one-fourth of all pediatric malignancies (2). The development of surgical interventions and contributions from various multicenter trials have helped in improving the survivability from a mere 40% in the early 1940s to more than 90% in the current times (9, 10). This profound accomplishment by the existential gold standard technique, i.e., radical nephrectomy (11), has directed the line of current research to elucidate measures which simultaneously aim to reduce postoperative complications while maintaining the survival rate (12, 13).

Recent literature has recommended the use of nephron sparing surgery as a possible alternative to the conventional radical nephrectomy approach (12–15). Predominantly, the use of nephron sparing surgery has garnered attention owing to its ability to reduce postoperative complications as compared to radical nephrectomy (12, 16). The technique has been reported to preserve long-term renal function, cardiovascular functionality (17), and better cosmesis (10), and prevent relapse (12). Lopes et al. (16), for instance, reported that the use of radical nephrectomy predisposed patients to a higher risk of renal failure [reduced estimated glomerular filtration rate (eGFR) rate] in the later phases of life, whereas the use of nephron sparing surgery did not (15). Likewise, negligible relapse has been reported for patients who underwent nephron sparing surgery as compared to those who had radical nephrectomy (15, 18).

Furthermore, nephron sparing surgery has been strongly advocated by the Children's Oncology Group (19, 20). The group supports its use in cases of unilateral Wilms tumor (<4 cm) where renal failure is imminent and/or when the onset of Wilms tumor begins in the unaffected kidney (19). Its use has also been backed by growing evidence from renal-cell carcinoma studies in adult population groups. Moreover, the preservation of renal function (i.e., reduced end stage renal disease) and overall health with nephron sparing surgery is extensively documented (18, 21, 22). Nevertheless, despite having several advancements over the conventional gold standard approach, concerns regarding the applicability of nephron sparing surgery still exist (23). Several studies have suggested a lack of volumetric, high-quality, multicenter data as a major reason for this backdrop (13, 21, 24). To date and to the best of our knowledge, no systematic review or meta-analyses has attempted to compare the effectiveness of these two surgical procedures for managing unilateral Wilms tumor. Such an attempt would help clinicians and researchers to determine a valid approach to manage unilateral Wilms tumor.

In this present systematic review and meta-analysis, we aim to address this substantial gap in the literature by comparing the efficiency of radical nephrectomy and nephron sparing surgery for managing unilateral Wilms tumor. We will compare the outcomes for these surgical interventions in terms of outcome for renal function, cancer relapse, and survivability.

## Materials and Methods

This systematic review and meta-analysis was carried in adherence to the Preferred Reporting Items for Systematic Reviews and Meta-Analyses (PRISMA) guidelines (25).

### Data Search Strategy

We searched four academic databases (MEDLINE, CENTRAL, EMBASE, and Scopus) from inception until November 2019 using MeSH keywords. We used the following terms in all academic databases in different combinations “Wilm's Tumor,” “Unilateral Wilm's Tumor,” “Unilateral Wilms' tumor,” “Tumor,” “Overall Survival,” “Pediatric Cancer,” “Nephron Sparing Surgery,” “Partial Nephrectomy,” “Radical Nephrectomy,” and “Total Nephrectomy.” In addition, we manually screened the bibliography of the included studies for any additional relevant study. The inclusion criteria for the included studies were as follows:

- Studies evaluated the efficacy of radical nephrectomy and/or nephron sparing surgery on unilateral Wilms tumor.
- Studies evaluated pediatric population, i.e., <18 years old.
- Studies evaluated survival rate and/or renal disease insufficiency [e.g., eGFR, end-stage renal disease (ESDR) outcome ratio].
- Studies were either randomized controlled trials, quasi-randomized controlled trials, controlled clinical trials, prospective observational trials with control groups, or retrospective trials.
- Studies published in peer-reviewed scientific journals and conferences.
- Studies published in the English language.

The selection procedure was independently replicated by two reviewers to avoid biasing. The following data were extracted from the included studies: authors, sample description (gender, age, health status), tumor classification, comorbidities, surgical intervention, and outcome measures. In the articles where quantitative data outcomes were not mentioned, the reviewers made attempts to contact respective corresponding authors for additional data.

### Quality Assessment

The risk of bias in the included studies was assessed by Cochrane's risk of bias assessment tool for randomized controlled trials and non-randomized controlled trials, i.e., Risk of Bias in Non-randomized Studies of Interventions (ROBINS-I) (26). The included studies were independently appraised by two reviewers. Inadequate randomization, concealment of allocation, confounding bias, and reporting of selective outcomes were considered as major threats for biasing (27). In cases of ambiguity, discussions were held between the reviewers until a consensus was reached. Moreover, a level of evidence analysis based on center for evidence-based medicine was also included (28).

### Data Analysis

A meta-analysis of the included studies was carried out using Comprehensive Meta-analysis version 2.0 (CMA) (29). The data were distributed and separately analyzed for survival rate, relapse rate, and renal function, i.e., estimated glomerular filtration rate. A meta-analysis was conducted based on the random effects model (30). The effect sizes are reported as weighted Hedge's *g*. The threshold for interpreting the weighted effect sizes are ≤ 0.2 a small effect, ≤ 0.5 as a medium effect, and ≥0.8 a large effect (31). Heterogeneity was assessed by computing *I*^2^ statistics. The threshold for interpreting heterogeneity is 0–25% with negligible heterogeneity, 25–75% with moderate heterogeneity, and ≥75% with substantial heterogeneity (32). Sensitivity analyses were performed in cases where potential sources of heterogeneity existed (33). Here, based on the presence or absence of inadequate randomization methods in the studies, we either included or excluded the results of the studies. For each evaluated parameter details of weighted effect size, 95% confidence intervals, level of significance, and heterogeneity have been duly reported. The publication bias was not investigated due to the limited number of studies (i.e., <10) that reported each outcome. The alpha level was set at 5%.

## Results

**Figure 1:**
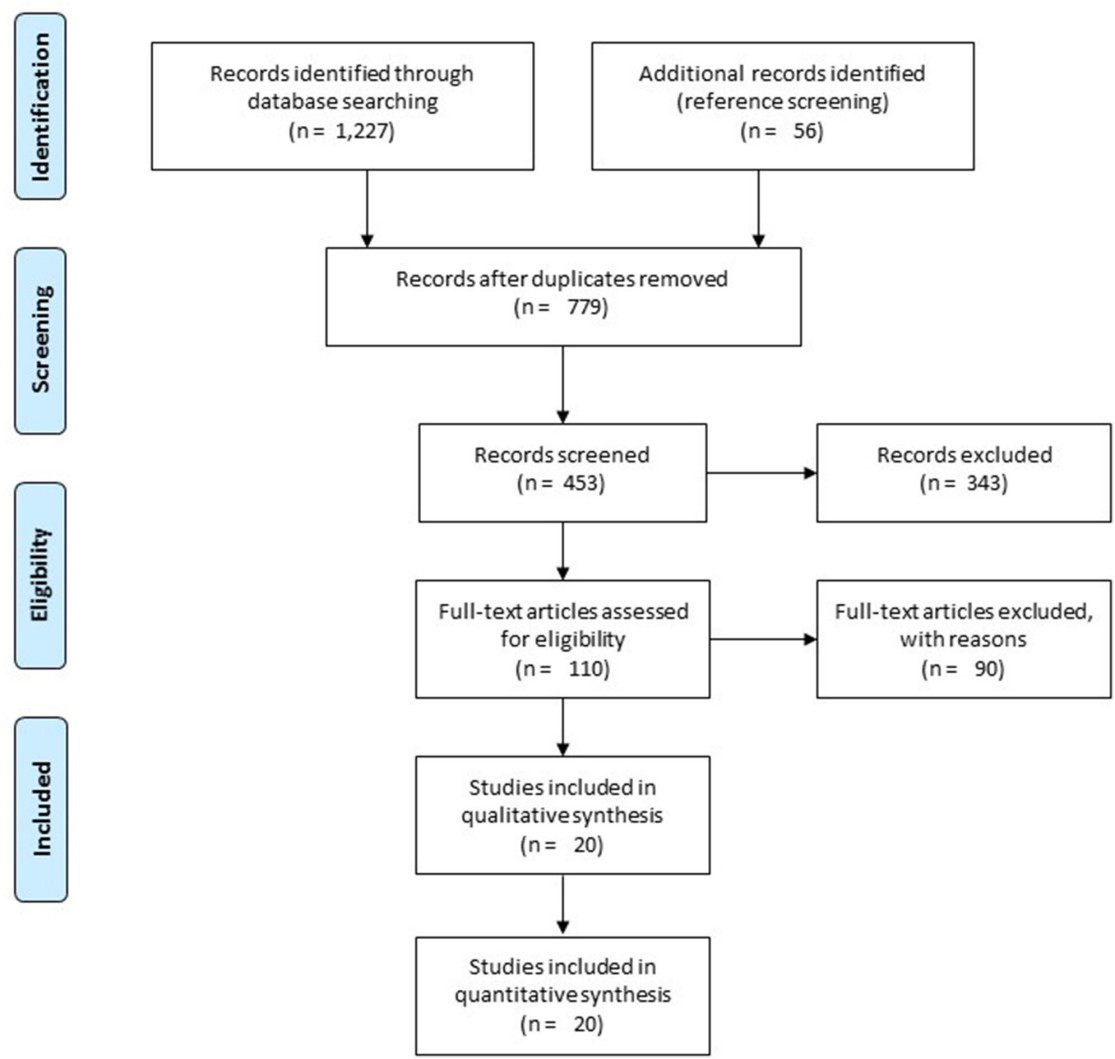
Illustrates the Preferred Reporting Items for Systematic Reviews and Meta-Analyses (PRISMA) flow chart for the included studies.

A preliminary search on four academic databases resulted in a total of 1,227 studies; 56 more studies were included after the bibliography of articles were screened (Figure 1). Thereafter, upon excluding the duplicates and applying the inclusion criteria, a total of 20 studies were retained. Thirteen of the included studies were retrospective cohort studies (12, 15, 34–43), whereas seven of the included studies were prospective cohort studies (44–50). Qualitative and quantitative data were then extracted from all the studies and summarized in Table 1.

**Table 1:** Illustrates the characteristics of the included studies.

**References**	**Age (*M* ± SD)**	**Sample size (female, male)**	**Tumor size (stage)**	**Assessment**	**Follow-up mean (SD or range)**	**Surgical intervention**	**Subtype of surgical intervention**
Ceccanti et al. (49)	3.3 ± 3.2 years	12 (8F, 4M)	6.3 ± 3.4 cm (T1: 1, T2: 2, T3: 9, T4: 0, T5)	Estimated glomerular filtration rate	5, 10 years and last follow-up (17.3 ± 7.6 years)	Zero-ischemia NSS	Enucleation: 4 Partial nephrectomy: 8
Spiegl et al. (50)	NSS: 2.3 ± 1.5 years RN: 4.6 ± 3.7 years	NSS: 9 (NR) RN: 54 (33F, 21M)	NSS: NR RN: NR (T1: 15, T2: 12, T3: 27, T4: 0, T5: 0)	Survival rate, relapse and renal insufficiency rate	NSS: 2.9 years RN: 4.1 years	NSS, RN	–
Mor et al. (43)	2.5 (0.5–3.7) years	35 (17F, 19M)	10 cm	Estimated glomerular filtration (serum creatinine level) and survival rate	–	RN	–
Interiano et al. (48)	3.2 (0.2–12.1) years	75 (44F, 31M)	NR (TI: 41, TII: 33, TIII: 1, TIV: 0, TV: 0)	Estimated glomerular filtration rate	19.6 (10–32.8) years	RN	–
Nerli et al. (42)	1.6 ± 1.1 years	9 (NR)	6.5 ± 2.5 cm (T1: 15, T2: 12, T3: 27, T4: 0, T5: 0)	Estimated glomerular filtration (creatinine clearance rate)	1.8 ± 0.9 years	NSS	Heminephrectomy: 2 Partial nephrectomy: 7
Wilde et al. (47)	–	NSS: 91 RN: 2,709	NSS: NR (TI: 59, TII: 13, TIII: 12, TIV: 0, TV: 0) RN: NR (TI: 1,294, TII: 638, TIII: 712, TIV: 0, TV: 0)	Survival and relapse rate	NS: 3 (2.5–4.2) years RN: 4 (3.9–4.2)	NSS, RN	–
Wang et al. (12)	NSS: 3.2 ± 2.9 years RN: 3.2 ± 2.8	NSS: 114 (65F, 49M) RN: 1,720 (893F, 827M)	NSS: 9 ± 7.9 cm RN: 11 ± 5.4 cm	Survival rate	NSS: 5.3 ± 6.5 RN: 7.2 ± 10.1 years	NSS, RN	–
Cost et al. (37)	NSS: 2.5 ± 3.6 years RN: 3.7 ± 3.1 years	NSS: 15 (6F, 9M) RN: 15 (8F, 7M)	NSS: NR (T1: 10, T2: 2, T3: 3, T4: 0, T5: 0) RN: NR (T1: 10, T2: 2, T3: 3, T4: 0, T5: 0)	Estimated glomerular filtration rate and survival rate	NSS: 8.4 ± 7.9 years RN: 2.1 ± 5.6 years	NSS, RN	–
Cost et al. (36)	–	121	NR (TI: 24, TII: 45, TIII: 29, TIV: 23, TV: 0)	Survival and relapse rate	5.7 (0.1–17.8) years	RN	–
Kern et al. (41)	Normal GFR: 2.7 ± 2.3 years Abnormal GFR: 5.6 ± 7.2 years	55 (33F, 22M) Normal GFR: 43 Abnormal GFR: 8	Normal GFR: NR Abnormal GFR: NR	Estimated glomerular filtration rate (creatinine clearance)	Normal GFR: 7.3 ± 4.5 years Abnormal GFR: 11.4 ± 4.3 years	RN	–
Cozzi et al. (39)	NSS: 3.9 ± 3.2 years RN: 3.6 ± 2.9 years	NSS: 12 (8F, 4M) RN: 42 (24F, 18M)	NSS: NR (T1: 12, T2: 0, T3: 0, T4: 0, T5: 0) RN: NR (T1: 27, T2: 0, T3: 15, T4: 0, T5: 0)	Estimated glomerular filtration rate and survival rate	NSS: 11.7 ± 6.5 years RN: 11.3 ± 7.8 years	NSS, RN	–
Sanpakit et al. (40)	3 ± 2 years	30 (14F, 16M)	NR (T1: 3, T2: 11, T3: 8, T4: 5, T5: 0)	Estimated glomerular filtration rate, survival and relapse rate	4.76 (0.1–13.9) years	RN	–
Romão et al. (15)	2.2 ± 2.7 years	8 (NR)	8.6 ± 8.7 cm (T1: 5, T2: 0, T3: 1, T4: 0, T5: 0)	Estimated glomerular filtration rate (creatinine clearance)	3 ± 1.8 years	NSS	Radical: 3 Partial nephrectomy: 6
Cozzi et al. (46)	NSS: 3.5 ± 3.5 years RN: 4.6 ± 3.4 years	NSS: 10 (7F, 3M) RN: 15 (9F, 6M)	NSS: NR (T1: 10, T2: 0, T3: 0, T4: 0, T5: 0) RN: NR (T1: 6, T2: 3, T3: 3, T4: 3, T5: 0)	Estimated glomerular filtration rate (creatinine clearance)	NSS: 12.3 ± 4 years RN: 12.3 ± 4 years	NSS, RN	–
Szymik-Kantorowicz et al. (38)	NSS: 1.2 ± 0.7 years RN: 5 ± 2.64 years	NSS: 6 (NR) RN: 3 (NR)	NSS: NR (T1: 6, T2: 0, T3: 0, T4: 0, T5: 0) RN: NR (T1: 3, T2: 0, T3: 0, T4: 0, T5: 0)	Survival rate and relapse	NSS: 4.2 ± 2.83 years RN: 3.3 ± 1.53 years	NSS, RN	–
Daw et al. (44)	5.4 ± 3.9 years	12 (9F, 3M)	NR (T1: 0, T2: 1, T3: 4, T4: 6, T5: 1)	Estimated glomerular filtration rate, survival and relapse rate	11.9 years	RN	–
Varlet et al. (45)	3.3 ± 3.2 years	3 (2F, 1M)	RN: 8.5 ± 6.3 cm (TI: 0, TII: 0, TIII: 0, TIV: 3, TV: 0)	Survival rate and relapse	1.5 (1–2.6) years	Laparoscopic RN	–
Zani et al. (51)	3.2 ± 0.4 years	NSS: 10 RN: 40 52 (37F, 15M)	NR (T1: 27, T2: 23, T3: 2, T4: 0, T5: 0)	Survival rate and relapse	15.7 years	NSS, RN	–
Linni et al. (34)	4 ± 2.8 years	11 (8F, 3M)	NR (T1: 4, T2: 0, T3: 1, T4: 2, T5: 4)	Estimated glomerular filtration (creatinine clearance) and survival rate	6.6 years	NSS	Enucleation: 4, Partial nephrectomy: 7
Duarte et al. (35)	3.6 ± 1.75 years	8 (4F, 4M)	9.7 ± 5.4 cm	Relapse rate	(0.4–1.9) years	Laparoscopic RN	–

*F, female; M, male; NSS, nephron sparing surgery; RN, radical nephrectomy*.

### Participant Information

A total of 5,246 children with unilateral Wilms tumor were evaluated in the studies included in this review. All the studies included a mixed gender population, i.e., 1,229 girls and 1,062 boys. However, five studies did not mention the gender of their evaluated sample (15, 37, 38, 42, 47). Two hundred ninety-seven children with unilateral Wilms tumor were operated by nephron sparing surgery, whereas 4,897 children were treated by radical nephrectomy. One study compared the effects between the two surgical interventions but did not specify the distribution of the sample (51). Likewise, the average duration of follow-up was 7 ± 4.7, 7.4 ± 5.1 years for children operated with nephron sparing surgery and radical nephrectomy, respectively. Two studies did not explicitly specify the duration of follow-up (43, 51).

### Risk of Bias

**Figure 2:**
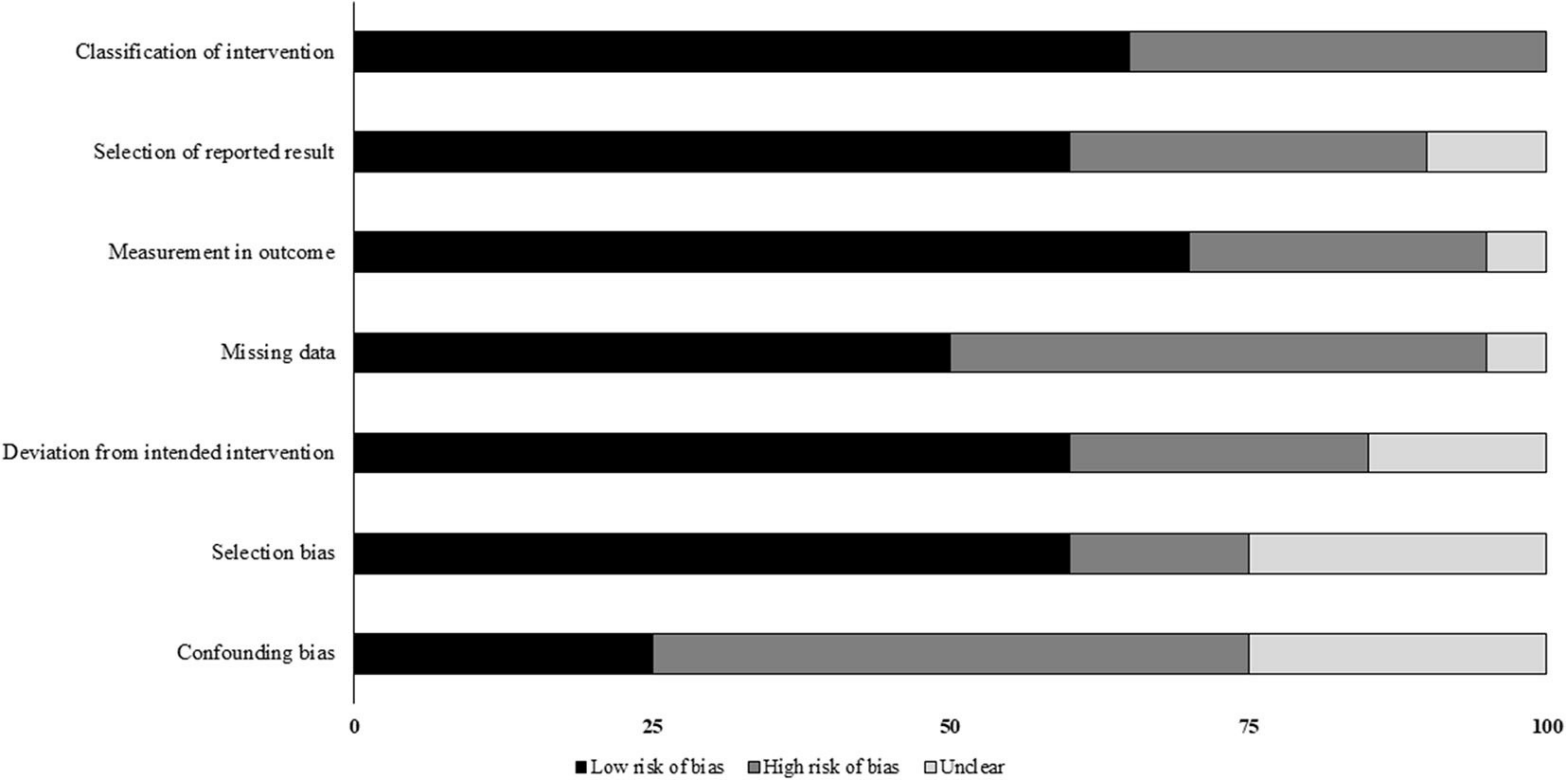
Illustrates risk of bias within studies according to Risk of Bias in Non-randomized Studies of Interventions (ROBINS-I) scale (x-axis: %).

The prevalence of risk of bias according to Cochrane's risk of bias assessment tool for non-randomized controlled trials ROBINS-I has been demonstrated in Table 2 and Figure 2. The overall risk in the included studies is high. The highest risk of bias was observed to be due to the lack of clarity in the confounding factors and the missing data. Furthermore, the studies refrained from explaining the measures they undertook to manage missing data and/or analyses for intention to treat analysis. A level of evidence of 2b was observed for all the included studies based on their experimental design.

**Table 2:** Illustrates the quality of the analyzed studies according to the Cochrane risk of bias assessment tool for non-randomized controlled trials ROBINS-I.

**References**	**Design**	**Confounding bias**	**Selection bias**	**Deviation from intended intervention**	**Missing data**	**Measurement in outcome**	**Selection of reported result**	**Classification of intervention**	**Level of evidence**
Ceccanti et al. (49)	PCS	?	+	+	+	–	–	+	2b
Spiegl et al. (50)	PCS	–	?	+	–	+	+	+	2b
Mor et al. (43)	RCS	–	?	+	–	+	+	+	2b
Interiano et al. (48)	PCS	+	+	–	–	+	–	+	2b
Nerli et al. (42)	RCS	+	+	–	+	–	+	+	2b
Wilde et al. (47)	PCS	?	+	+	+	+	–	+	2b
Cost et al. (37)	RCS	+	+	?	+	+	+	+	2b
Wang et al. (12)	RCS	+	+	+	+	+	?	+	2b
Cost et al. (36)	RCS	–	+	+	–	–	+	–	2b
Kern et al. (41)	RCS	–	?	+	+	+	+	+	2b
Cozzi et al. (39)	RCS	–	+	?	–	–	+	–	2b
Sanpakit et al. (40)	RCS	?	+	–	–	+	–	–	2b
Romão et al. (15)	RCS	–	+	+	+	+	+	+	2b
Cozzi et al. (46)	PCS	–	+	+	+	+	+	–	2b
Szymik-Kantorowicz et al. (38)	RCS	?	–	+	–	?	–	+	2b
Daw et al. (44)	PCS	–	–	+	+	+	+	+	2b
Varlet et al. (45)	PCS	–	+	–	+	–	?	–	2b
Zani et al. (51)	RCS	?	–	?	–	+	+	–	2b
Linni et al. (34)	RCS	+	?	+	–	+	+	+	2b
Duarte et al. (35)	RCS	–	?	–	?	+	–	–	2b

*PCS, prospective cohort study; RCS, retrospective cohort study; –, high risk of bias; +, low risk of bias; ?, unclear risk of bias*.

### Survival Rate

The survival rate was compared in six studies. Here, 10 studies reported the survival rate of children operated with radical nephrectomy (12, 37–40, 43–45, 47, 50), whereas six studies reported the outcome after nephron sparing surgery (12, 37–39, 47, 50). A mean survival rate of 90.6 ± 8.7% was reported in 4,623 children operated with radical nephrectomy during a mean follow-up of 5.9 ± 3.5 years. A higher mean survival rate of 95.1 ± 5.9% was reported in 293 children operated with nephron sparing surgery during a mean follow-up of 5.9 ± 3.4 years.

### Relapse Rate

Mean relapse rate was reported in 10 studies. Here, eight studies reported the survival rate of children operated with radical nephrectomy (35, 37, 38, 40, 44, 45, 47, 50), whereas two studies reported the outcome after nephron sparing surgery (38, 47). A mean relapse rate of 8 ± 8.8% was reported in 2,940 children operated with radical nephrectomy during a mean follow-up of 4.4 ± 3.4 years, whereas a mean relapse rate of 2 ± 2.8% was reported in 97 children operated with nephron sparing surgery during a mean follow-up of 3.6 ± 0.8 years.

### Estimated Glomerular Filtration Rate

#### Nephron Sparing Surgery

Seven studies assessed estimated glomerular filtration rate for cases operated with nephron sparing surgery (15, 34, 37, 39, 42, 46, 49), at a mean follow-up of 8.7 ± 5.4 years. A mean estimated glomerular filtration rate of 93.9 ± 27.5 ml/min per 1.73 m^2^ was reported before the operation and 116.2 ± 27.4 ml/min per 1.73 m^2^ after the operation.

#### Radical Nephrectomy

Eight studies assessed estimated glomerular filtration rate in cases operated with radical nephrectomy (37, 39–41, 43, 44, 46, 48), at a mean follow-up of 10.6 ± 4.9 years. A mean estimated glomerular filtration rate of 104.2 ± 24.3 ml/min per 1.73 m^2^ was reported before the operation and 98.3 ± 17.8 ml/min per 1.73 m^2^ after the operation.

### Meta-Analysis Reports

#### Survival Rate

**Figure 3:**
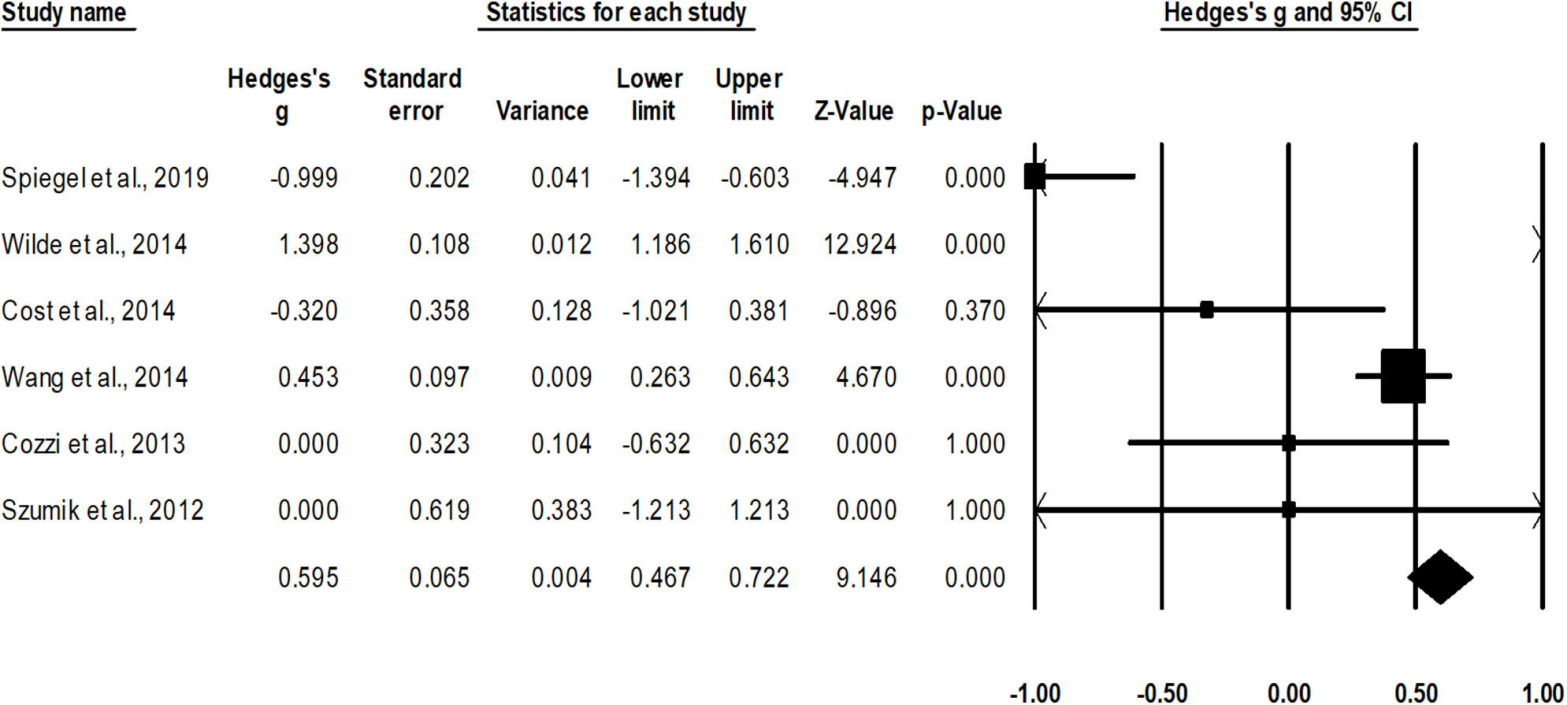
Illustrates the forest plot for studies evaluating the effects of nephron sparing surgery on survival rate in children with unilateral Wilms tumor. Weighted effect size is presented as boxes; 95% CIs are presented as whiskers. They demonstrate survival rate (%) in between cases operated with radical nephrectomy and nephron sparing surgery. A negative effect represents enhanced survival for radical nephrectomy; a positive effect represents enhanced survival for nephron sparing surgery.

Survival rate was assessed in six studies (12, 37–39, 47, 50). An across group, random-effect analysis (Figure 3) revealed a *medium* significant positive effect of nephron sparing surgery on survival rate as compared to radical nephrectomy (*g*, 0.59; 95% CI, 0.46–0.72, *p* < 0.001) with no heterogeneity (*I*^2^, 0%).

#### Relapse Rate

**Figure 4:**
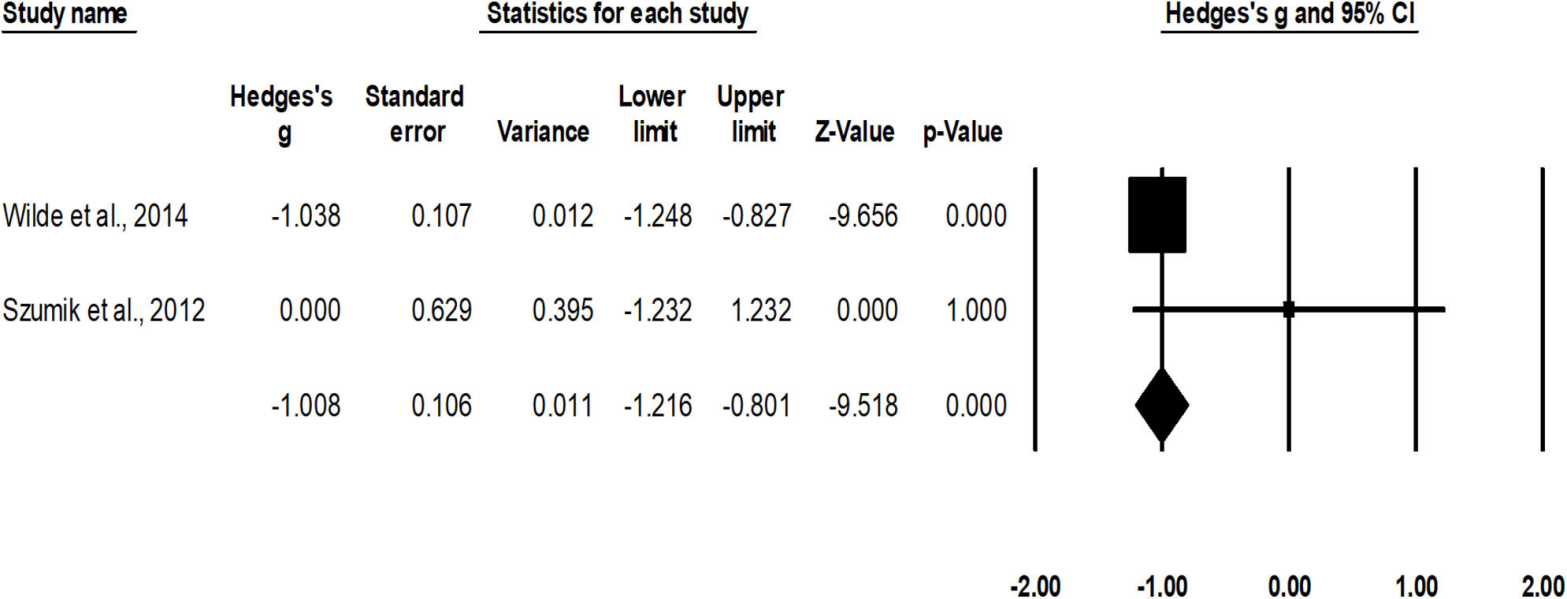
Illustrates the forest plot for studies evaluating the effects of nephron sparing surgery on occurrence of relapse in children with unilateral Wilms tumor. Weighted effect size is presented as boxes; 95% CIs are presented as whiskers. They demonstrate relapse rate (%) in between cases operated with radical nephrectomy and nephron sparing surgery. A negative effect represents a reduced occurrence of relapse for nephron sparing surgery; a positive effect represents reduced occurrence of relapse for radical nephrectomy.

Rate of relapse was assessed in two studies (38, 47). An across-group, random-effect analysis (Figure 4) revealed a *large* significant negative effect of nephron sparing surgery to reduce the occurrence of relapse compared to radical nephrectomy (*g*, −1.0; 95% CI, −1.2 to −0.8; *p* < 0.001) with no heterogeneity (*I*^2^, 0%).

### Estimated Glomerular Filtration Rate

#### Nephron Sparing Surgery

**Figure 5:**
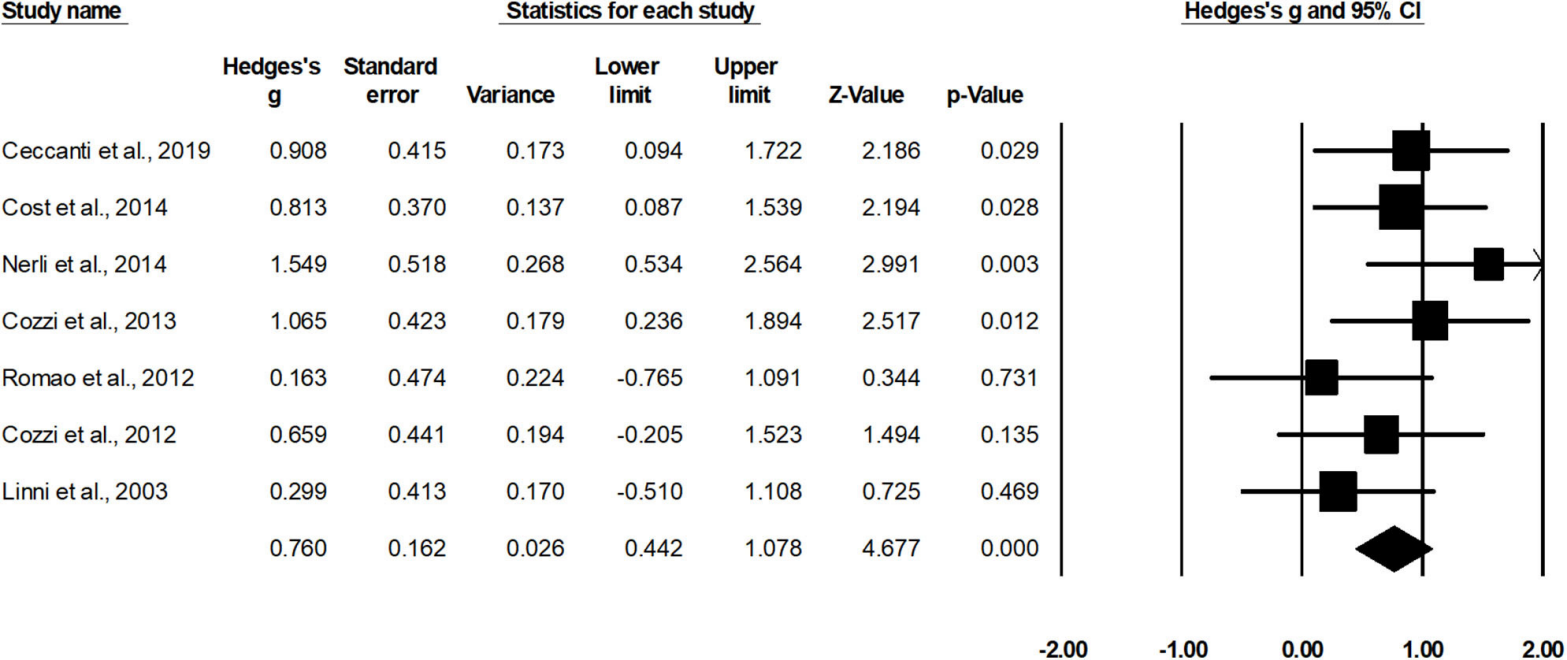
Illustrates the forest plot for studies evaluating the effects of nephron sparing surgery on estimated glomerular filtration rate in children with unilateral Wilms tumor. Weighted effect size is presented as boxes; 95% CIs are presented as whiskers. They demonstrate estimated glomerular filtration rate (ml/min per 1.73 m^2^) before and after the surgical intervention. A negative effect represents a reduced glomerular filtration rate; a positive effect represents enhanced glomerular filtration rate.

Seven studies assessed estimated glomerular filtration rate for cases operated with nephron sparing surgery (15, 34, 37, 39, 42, 46, 49). A within-group, random-effect analysis (Figure 5) revealed a *medium* significant effect of nephron sparing surgery on estimated glomerular filtration rate in positive domain (*g*, 0.76; 95% CI, 0.44–1.07; *p* < 0.001) with no heterogeneity (*I*^2^, 0%).

#### Radical Nephrectomy

**Figure 6:**
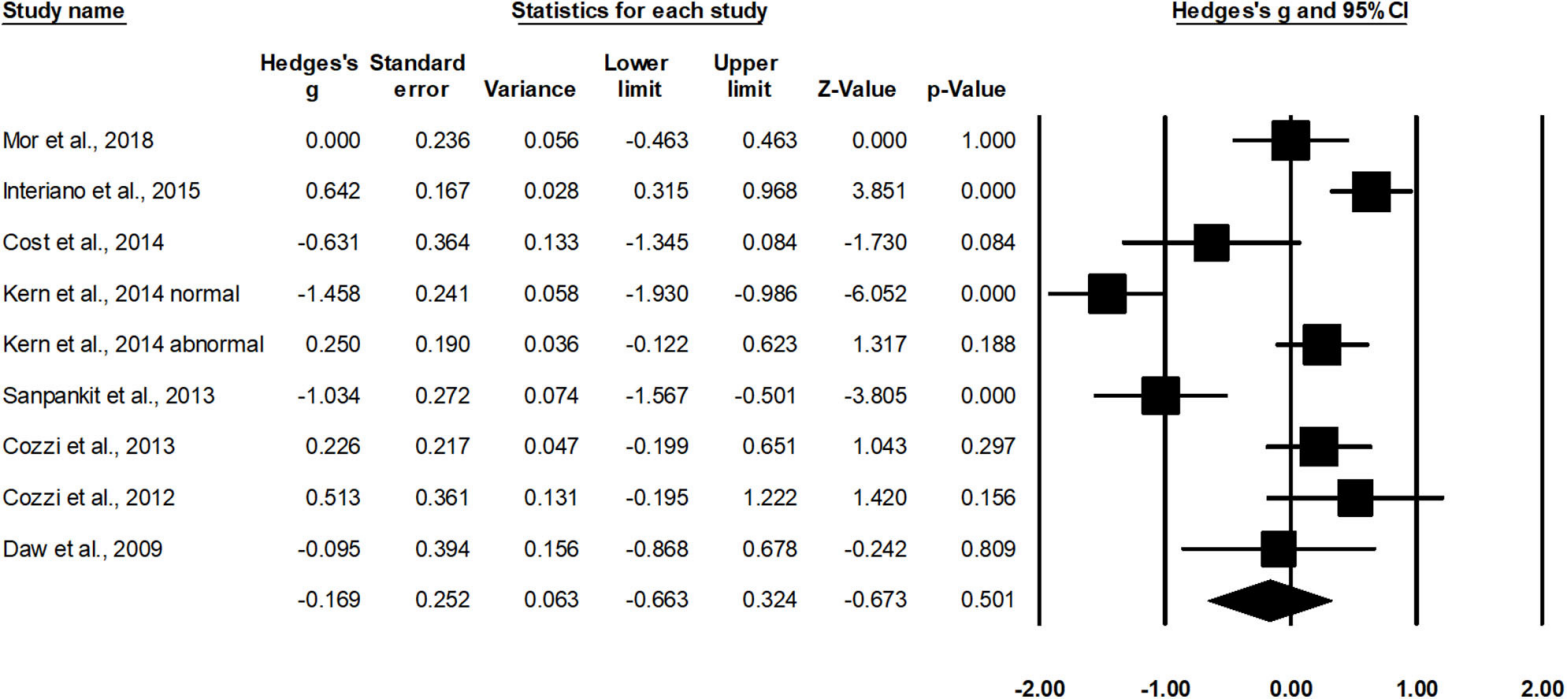
Illustrates the forest plot for studies evaluating the effects of radical nephrectomy on estimated glomerular filtration rate in children with unilateral Wilms tumor. Weighted effect size is presented as boxes; 95% CIs are presented as whiskers. They demonstrate estimated glomerular filtration rate (ml/min per 1.73 m^2^) before and after the surgical intervention. A negative effect represents a reduced glomerular filtration rate; a positive effect represents enhanced glomerular filtration rate.

Eight studies assessed estimated glomerular filtration rate in cases operated with radical nephrectomy (37, 39–41, 43, 44, 46, 48). Additional subgroup data regarding groups with initially normal or abnormal levels of estimated glomerular filtration were extracted from one study (41). The random-effect analysis (Figure 6) revealed a *small* non-significant effect of radical nephrectomy on estimated glomerular filtration rate in negative domain (*g*, −0.16; 95% CI, −0.66–0.32; *p* = 0.50) with no heterogeneity (*I*^2^, 0%).

## Discussion

This present meta-analysis is the first to compare and quantify the beneficial effects of nephron sparing surgery over radical nephrectomy to manage unilateral Wilms tumor. We report a beneficial effect of nephron sparing surgery on survivability, renal function, and reduced relapse as compared to radical nephrectomy.

In the past decades, radical nephrectomy has been the choice of treatment for managing unilateral Wilms tumor (52, 53). The ability of this gold standard approach has been favored because of its ability to limit positive margins (52), spillage (43), residual tumor (13), and recurrence (53). Nevertheless, its ability to limit renal function as a result of substantial resection of unaffected renal tissue has raised questions concerning its efficiency (24, 54, 55). In addition, nephrotoxic effects of cancer therapies, genetic predisposition (WT1 mutation), and hyperfiltration injury have also been reported to contribute toward worsening renal function. Cost et al. (37) for instance, reported a reduction in renal function in children operated with radical nephrectomy after a mean follow-up of 2.1 years. Likewise, Kern et al. (41) and Cozzi et al. (39) reported a reduction in glomerular filtration rate after radical nephrectomy. The authors mentioned an inverse relationship between a longer follow-up and renal function postsurgery. In the present meta-analysis, we too observed that the renal function was negatively affected (eGFR, −0.16; mean follow-up, 10.6 years) in cases undergoing radical nephrectomy.

In order to counteract these detrimental effects, the use of nephron sparing surgery as a substitution for managing unilateral Wilms tumor has garnered a lot of attention (12, 18, 24, 56). The intervention has been reported to spare the maximum amount of unaffected kidney tissue to preserve renal function, which in turn has been associated with better health status outcomes (57, 58). Antonelli et al. (59), for instance, reported an independent relationship between cancer-specific mortality and renal function (estimated glomerular filtration rate). From the studies included in this review, Nerli et al. (42) reported that an intervention by nephron sparing surgery was potent enough to preserve kidney function (creatinine clearance) during a shorter follow-up of 1.8 years. For prolonged follow-ups, Ceccanti et al. (49) demonstrated that both renal function (99mTcdimercapto succinic acid renal scintigraphy) and renal outcome (ultrasonography) were enhanced in their cohort after a zero-ischemic nephron sparing surgery. The authors reported these enhancements in follow-up screening performed after 5, 10, and 17.3 years (49), respectively. In the current meta-analyses, we too observed *large* enhancements (eGFR, 0.76; follow-up, 8.7 years) in renal function outcome in children operated with nephron sparing surgery. According to the literature, this enhancement in renal functioning can further enhance overall health status by alleviating cardiovascular functioning (60), and quality of life (57).

Additionally, discrepancies in terms of recurrence and survivability have also been reported between the two approaches (21, 24, 47). For instance, Wilde et al. (47) in a retrospective trial of 2,800 patients reported that tumor relapse occurred in 13% of the cases operated with radical nephrectomy as compared to a mere 4% being treated with nephron sparing surgery. The authors, however, did not report any difference in between the two approaches in terms of survivability (47). Cozzi et al. (39), on the contrary, compared survivability between the two approaches during a mean follow-up of 11.5 years. The authors reported that while the survival rate for cases operated with nephron sparing surgery was 100%, only 74% of the cases treated with radical nephrectomy survived. In this present review, we address this discrepancy and report beneficial effects of nephron sparing surgery on survivability (*g*, 0.59) as compared to radical nephrectomy. Likewise, we also report negative effects of nephron sparing surgery for reducing the occurrence of relapse (*g*, −1.0) as compared to radical nephrectomy. We believe that skillful application as demonstrated by high technical success rate of the surgery could be one of the reasons why nephron sparing surgery accounts for lesser tumor spillage and therefore lesser cases of relapse (61). Besides, in terms of higher survivability, we presume that preserving renal function could have positively impacted the overall survivability of the patient [for more details, see (59)]. In the present literature review, a few limitations persisted. A lack of statistical data in the included cohort studies can bias our interpretations concerning the influence of nephron sparing surgery over radical nephrectomy. For instance, the evaluation of relapse rate was performed only in two studies. Here, 2,712 cases were operated with radical nephrectomy, whereas only 97 cases were operated by nephron sparing surgery. Therefore, the outcome of a large effect suggesting a lower incidence of relapse could also possibly be a result of type II error (62). We recommend future studies to address this paucity of data by sharing descriptive statistics in open access databases for cases operated with nephron sparing surgery. Second, despite a broad inclusion criterion, we were able to include only prospective and retrospective cohort studies. Due to this, the present review reports a 2b level of evidence in support of nephron sparing surgery. Nonetheless, several of the included studies in the review, although supporting the use of nephron sparing surgery, also draw caution toward the interpretation of their results because of smaller sample size and poorer quality of studies (15, 21, 36, 58). Future studies are recommended to address this limitation by carrying out multicenter, double-blinded, randomized controlled trials to support the evidence.

## Conclusion

In conclusion, this systematic review and meta-analysis provides a 2b level of evidence to suggest the use of nephron sparing surgery to manage unilateral Wilms tumor. The findings from the current meta-analyses report higher survivability, higher levels of renal functioning, and lesser incidences of relapse postintervention by nephron sparing surgery.

## Data Availability Statement

Publicly available datasets were analyzed in this study. This data can be found here: MEDLINE, CENTRAL, EMBASE, and Scopus.

## Author Contributions

HC designed the paper and prepared the manuscript. HC, SY, and CQ were involved in literature search and data interpretation. SY were responsible for the data analysis. CQ edited the manuscript. All authors have read and approved the final manuscript.

## Conflict of Interest

The authors declare that the research was conducted in the absence of any commercial or financial relationships that could be construed as a potential conflict of interest.
